# Effect of ancient wheat pasta on gut microbiota composition and bacteria-derived metabolites: A randomized controlled trial

**DOI:** 10.3389/fnut.2022.971666

**Published:** 2022-08-04

**Authors:** Simone Baldi, Monica Dinu, Giuditta Pagliai, Barbara Colombini, Leandro Di Gloria, Lavinia Curini, Marco Pallecchi, Matteo Ramazzotti, Gianluca Bartolucci, Stefano Benedettelli, Amedeo Amedei, Francesco Sofi

**Affiliations:** ^1^Department of Experimental and Clinical Medicine, University of Florence, Florence, Italy; ^2^Department of Biomedical, Experimental and Clinical Sciences “Mario Serio”, University of Florence, Florence, Italy; ^3^Department of Neurosciences, Psychology, Drug Research and Child Health, University of Florence, Florence, Italy; ^4^Department of Agrifood Production and Environmental Sciences, University of Florence, Florence, Italy; ^5^Interdisciplinary Internal Medicine Unit, Careggi University Hospital, Florence, Italy; ^6^Unit of Clinical Nutrition, Careggi University Hospital, Florence, Italy

**Keywords:** gut microbiota, ancient wheat, modern wheat, SCFAs, MCFAs, pasta

## Abstract

**Background and aim:**

In recent years, many studies have suggested that ancient wheat products might have beneficial effects on cardiometabolic risk profile, but little is known about their effect on gut microbiota (GM). The aim of the present study was to evaluate whether a replacement diet with pasta made from ancient wheat (AD) could influence the GM composition and its metabolites’ production compared to a replacement diet with pasta made from modern wheat (CD).

**Methods:**

A randomized, double-blinded crossover trial with two intervention phases was conducted on 20 clinically healthy adults (9 females; 11 males; mean age 43.1 ± 12.5 years). Study participants were assigned to consume pasta made using semi-whole flour from organic wheat that was either from ancient or modern control wheat for 8 weeks in a random order. An 8-week washout period was implemented between the interventions. Stool samples were collected from all subjects at the beginning and at the end of each intervention period. GM composition, and short- (SCFAs) and medium- chain fatty acids (MCFAs) production was evaluated.

**Results:**

Dietary interventions did not produce significant diversity in the GM composition at higher ranks (phylum, class, order and family), but only at genus level. In detail, the AD significantly (adj. p < 0.05) changed the abundance of *Erysipelatoclostridium* spp., *Bacteroides_pectinophilus_group* spp., *CAG-873* spp., and *Holdemanella* spp. The CD significantly affected the abundance of *Akkermansia* spp., *CAG-873* spp., *Hungatella* spp., *Lachnospiraceae_UCG-008* spp., *NK4A214_group* spp., *Frisingicoccus* spp., *Megasphaera* spp., *Synergistes* spp., and *Tyzzerella* spp. Regarding the production of SCFAs and MCFAs, AD resulted in a significant increase of fecal acetic (+0.7%), isobutyric (+30.1%), 2-methylbutyric (+64.2%), and isovaleric (+22.5%) acids. On the other hand, CD resulted in increased levels of isobutyric (+71.4%), 2-methylbutyric (+116.2%), isovaleric (+99%), and valeric (+21.4%) acids, and a reduction of butyric (-31.6%) and hexanoic (-66.4%) acids.

**Conclusion:**

A short-term replacement diet with both ancient and modern wheat pasta determined significant changes in GM composition at the genus level but notably the AD resulted in a greater beneficial impact on anti-inflammatory SCFAs.

## Introduction

Wheat (*Triticum spp.*), the second most produced cereal grain after maize, currently represents the main staple food in many countries, providing a fifth of dietary calories and protein in human diets globally (1). In addition to being an important source of micronutrients, fiber and beneficial phytochemicals, several epidemiological studies have demonstrated an association between whole-wheat consumption and reduced risk of non-communicable diseases (*e.g.*, cardiovascular disease, type 2 diabetes, and some cancers, including colorectal and pancreatic)(2, 3).

Nevertheless, in the last years, many concerns have been raised about grain products because of their potential association with increased cases of food allergy and intolerances, especially autoimmune responses to gluten like celiac disease and related conditions (dermatitis herpetiformis and gluten ataxia), non-celiac gluten (orwheat) sensitivity, and irritable bowel syndrome associated with non-digestible carbohydrates (4–6). Therefore, ancient wheat cultivars (e.g., einkorn, spelt, emmer, Khorasan) have recently been in the spotlight as several clinical studies have suggested that they may represent a healthier choice than modern ones. The main explicative reasons are the reduced presence of immunoreactive products, with fewer amounts and types of reactive prolamins and fructans, and the higher content of minerals and nutraceutical compounds (7, 8).

Although recent studies have documented the beneficial effects of consuming ancient wheat products on pro-inflammatory/antioxidant parameters, as well as on cardiometabolic risk profile and fibromyalgia symptomatology (9) few studies have evaluated their effect on gut microbiota (GM) (8, 10). GM is involved in several metabolic processes and its susceptibility to the general food quality and the specific quality of wheat is well established (11). In particular, considering that GM plays a pivotal role in gut-associated immune system homeostasis, it has been demonstrated that wheat peptides, only partially digested by intestinal proteases, can have an increased or reduced immunogenicity depending on their degrading bacteria, influencing the possibility of developing wheat-related disorders (12). Another core GM activity is carbohydrate fermentation with the consequent production of short- (SCFAs) and medium-chain fatty acids (MCFAs), critical metabolites that can regulate intestinal physiology and modulate immune functions, as well as serving as energy source for colonocytes or other resident bacteria (13).

Therefore, the aim of the present study was to examine whether a replacement diet with pasta made with ancient wheat (AD) could influence the GM composition and its metabolite profile compared to a replacement diet with pasta made from modern wheat (CD).

## Materials and methods

### Study participants

All the participants were recruited from the Unit of Clinical Nutrition of the Careggi University Hospital, Florence, Italy. The study population was comprised of 20 volunteers (9 females; 11 males). Inclusion criteria to participate in the study were being between 18 and 65 years of age, being in good general health, and not having gastrointestinal disorders (e.g., chronic constipation, diarrhea, inflammatory bowel disease, irritable bowel syndrome, or other chronic gastrointestinal disorders). Subjects were excluded if they were taking medications or probiotics and similar (prebiotics or symbiotics) for any reason, were pregnant or lactating, was affected by celiac disease and non-celiac gluten (or wheat) sensitivity or had a serious illness or unstable condition. The study procedures were approved by the Ethics Committee of the Tuscany Region, Careggi University Hospital and followed the principles of the Declaration of Helsinki. Written informed consent was obtained from all participants before the start of the trial.

### Wheat varieties

The ancient wheat used in the present study was organic Evoldur1, an evolutionary population of durum wheat obtained by crossing 10 ancient Sicilian varieties: Russello, Ruscia, Tunisina, Scavuzza, Urria, Inglesa, Scorza Nera, Crotone, Chiattulidda, and Bidì and two turanic wheat varieties from the USDA collection (PI 125351 and PI 337643). The modern wheat used as control was organic ZetaE. The wheat varieties were grown at four farms that participated in the Tuscany Region-funded GrantSoilBiofert project, aimed at identifying agronomic techniques and selection models to obtain wheat suitable for the soil and climatic conditions of Tuscany (Sottomisura 16.2 - RDP 2014-2020). The farms were Azienda agricola Cini Francesco - il poderino di Asciano (Asciano, Siena), Azienda agricola Vecchioni Giovanna - Tenuta il tesorino (Follonica, Grosseto), Azienda agricola il Sorbo (Granaione, Grosseto), and Azienda agricola Tenute di Fraternita (Pieve al Toppo, Arezzo). The Pastificio Artigiano FABBRI s.a.s. (Strada in Chianti, Firenze) prepared the pasta (with no additives) from both the ancient and modern semolina, according to the artisan manufacturing procedures. To standardize the comparison, all the transformation procedures were identical for both the ancient and the modern wheat.

### Study design

The study was a randomized, double-blinded crossover trial with two intervention phases each lasting 8 weeks. After a run-in period of 2-weeks, the participants were divided into two groups which were respectively assigned to consume either ancient wheat (AD group) or modern wheat pasta (CD group) in a random order. An 8-week washout period was implemented between the interventions, in which participants were allowed to eat all foods according to their usual eating habits. In the second intervention period, the group assigned to consume the ancient wheat pasta in the first intervention period was assigned to consume the modern wheat pasta, and *vice versa.* Participants in both groups received 800 g per week of pasta, with no labels attached to the packages, and were informed that the products were organic and prepared by artisan methods. They were instructed to exclude other type of pasta from their respective diets, which was then “replaced” by ancient or modern pasta during the intervention phases. They were also advised to eat the pasta according to their normal consumption habits, and not to alter their dietary or lifestyle habits.

Both at the beginning and at the end of each intervention period, study participants were evaluated through objective examinations. General information about demographics, personal medical history and use of antibiotics or probiotics in the previous six months were collected. Weight and height were measured using a stadiometer. Body mass index (BMI) was calculated as the weight (kg)/height (m^2^). Stool samples were collected at the beginning and at the end of each intervention period to evaluate GM composition, SCFAs and MCFAs production.

### Gut microbiota characterization

Total DNA was extracted using the DNeasy PowerSoil Pro Kit (Qiagen, Hilden, Germany) from frozen (-80°C) stool samples, according to the manufacturer’s instructions. Briefly, 0.25 g of stool samples were added to a bead beating tube and homogenized with TissueLyser LT (Qiagen, Hilden, Germany) for 5min at 50 Hz. Afterward, DNA was captured on a silica membrane in a spin column format, washed and eluted. The quality and quantity of extracted DNA was assessed with both NanoDrop ND-1000 (Thermo Fisher Scientific, Waltham, MA, United States) and Qubit Fluorometer (Thermo Fisher Scientific, Waltham, MA, United States) and then it was frozen at -20°C. Subsequently, genomic DNA samples were sent to IGA Technology Services (Udine, Italy) where amplicons of the variable V3–V4 region of the bacterial 16S rRNA gene were sequenced in paired-end (2 × 300 cycles) on the Illumina MiSeq platform, according to the Illumina 16S Metagenomic Sequencing Library Preparation protocol.

Demultiplexed sequence reads were processed using QIIME2 2021.4 (14). The sequencing primers were removed using Cutadapt tool (15) while DADA2 (16) was used to perform paired-end reads merging, filtering and chimeras removal steps after trimming nucleotides from forward and reverse reads based on the quality profiles (–p-trunc-len-f 241 and –p-trunc-len-r 201). Hence, amplicon sequence variants (ASVs) were generated and the VSEARCH tool (17) was used for taxonomic assignment using the SILVA database (release 138) as reference, with a 0.99 identity threshold.

### Analysis of fecal SCFAs and MCFAs by gas chromatography-mass spectrometry

The qualitative and quantitative evaluation of fecal SCFAs and MCFAs was performed by Agilent gas chromatography-mass spectrometry (GC-MS) system composed with 5971 single quadrupole mass spectrometer, 5890 gas chromatograph and 7673 autosampler, through our previously described GC-MS method (18).

Briefly, just before the analysis, stool samples were thawed and added with sodium bicarbonate 0.25 mM solution (1:1 w/v) in a 1.5 mL centrifuge tube. Then, the obtained suspensions were sonicated for 5 minutes, centrifuged at 5000 rpm for 10 minutes and then the supernatants were collected. The SCFAs were finally extracted as follow: an aliquot of 100μL of sample solution (corresponding to 0.1 mg of stool sample) was added of 50 μL of internal standards mixture, 1 mL of tert-butyl methyl ether and 50 μL of HCl 6 M + 0.5 M NaCl solution in a 1.5 mL centrifuge tube. Subsequently, each tube was shaken in a vortex apparatus for 2 min, centrifuged at 10,000 rpm for 5 min, and lastly the solvent layer was transferred to an autosampler vial and processed three times.

### Statistical analysis

Statistical analyses on the bacterial communities were performed in R 4.1.0 with the help of the packages phyloseq 1.38.0, DESeq2 1.32.0 and other packages satisfying their dependencies as vegan 2.5-7. Packages ggplot2 3.3.5 and dendextend 1.15.2 were used to plot data and results. Data were reported as mean ± standard deviation (SD), number and percentage, or median percentage and interquartile range (IQR), as appropriate. Shannon index, Observed ASV richness and Pielou’s evenness were used to estimate bacterial diversity in each sample using the function estimate_richness from phyloseq. The Pielou’s evenness index was calculated using the formula E = S/log(R), where S is the Shannon diversity index and R is the number of ASVs in the sample. Differences in all indices were tested using the Wilcoxon test. PCoAs were performed on proportional count data of each sample, adjusted with square root transformation. At the different taxonomic ranks, the differential analyses of abundance were performed with DESeq2 on raw ASVs data (created using the tax_glom function in phyloseq). In addition, differential abundances of predicted pathways were determined and displayed using linear discriminant analysis (LDA) effect size (LefSe).

Furthermore, the software GraphPad Prism (v.5) was used for the statistical analysis of the fecal SCFAs’ and MCFA’s composition between pre- and post-AD samples and between pre- and post-CD samples. Pairwise differences between groups were assessed using Wilcoxon signed-rank test and p-values less than 0.05 were considered statically significant.

## Results

### Characteristics of the study population

**FIGURE 1:**
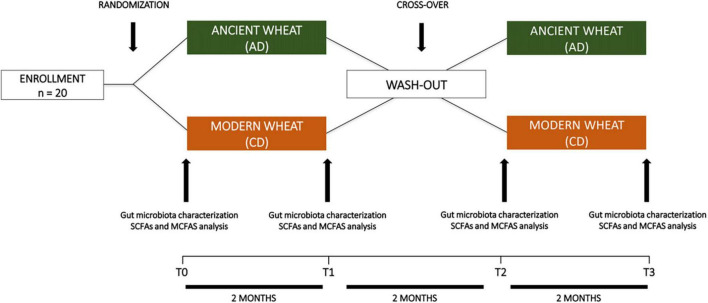
Workflow of the crossover intervention study.

The mean age of the study population was 43.1 ± 12.5 years. Body weight was 66.3 ± 14.1 kg, while BMI was 23.0 ± 2.7 kg/m^2^. As shown in Figure 1, **10** participants (6F; 4M) with a mean age of 46.8 ± 14.1 years and a BMI of 24.0 ± 3.0 kg/m^2^ were assigned to consume ancient wheat pasta (AD group), while the other ten participants (5F; 5M) with a mean age of 39.4 ± 10.0 years and a BMI of 22.0 ± 2.1 kg/m^2^ were assigned to consume modern wheat pasta (CD group). Subsequently, after the washout period, the study groups were crossed and, because of the retirement of two subjects, ten subjects (5F; 5M) consumed ancient wheat pasta and eight subjects (5F; 3M) consumed modern wheat pasta. No participants reported having used antibiotics or probiotics in the previous six months.

### GM composition

**FIGURE 2:**
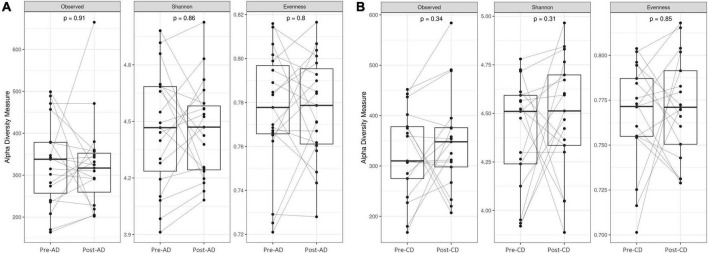
Box plots reporting alpha diversity indices (Observed ASV richness, Shannon index, Pielou’s evenness) pre- and post-AD **(A)** and pre- and post-CD **(B)**. Lines link paired samples and statistical differences were assessed using the Wilcoxon signed-rank test. *p*-values less than 0.05 were considered statistically significant. AD, ancient-wheat diet; CD, control-wheat diet.

First, we evaluated if the replacement diet with pasta made from ancient wheat and the replacement diet with pasta made from modern control wheat had an impact on the GM composition. As shown in Figure 2, no significant differences were reported on the different alpha diversity indices after both dietary interventions.

**FIGURE 3:**
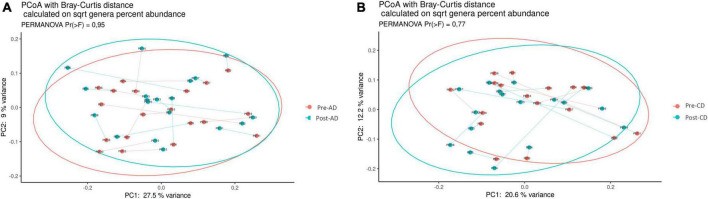
Principal coordinates analysis (PCoA), according to the Bray-Curtis beta-diversity metric, of pre- and post-AD samples **(A)** and pre- and post-CD samples **(B)**. Results of the permutational multivariate analysis of variance (PERMANOVA) are also shown based on the first two coordinates. AD, ancient-wheat diet; CD, control-wheat diet.

**FIGURE 4:**
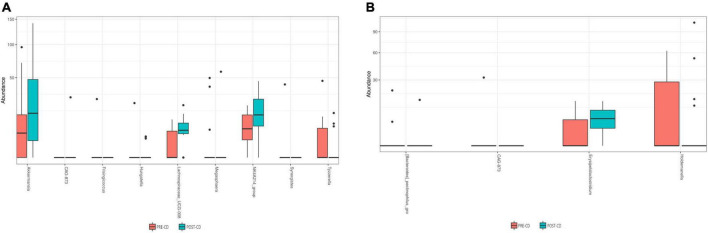
Box plots showing significant differentially abundant genera among pre- and post-AD **(A)** and pre- and post-CD **(B)** samples. AD, ancient-wheat diet; CD, control-wheat diet.

Similarly, beta diversity analysis, assessed through the Bray–Curtis dissimilarity metric, did not highlighted any clear separation at the genus level between the pre- and post-AD groups (Figure 3A) or among the pre- and post-CD groups (Figure 3B), neither at other taxonomic ranks. To evaluate the impact of both dietary interventions on microbial abundances, a taxonomic composition analysis was performed. While no significant changes were observed at phylum, class, order and family ranks, significant differences were observed at genus level. As shown in Figure 4A, AD determined a significant increase of *Erysipelatoclostridium* spp. (log2FC = 3.487; adj. *p* = 7.90e-4) and a significant decrease of *[Bacteroides]_pectinophilus_group* spp. (log2FC = -11.278; adj. *p* = 1.30e-4), *CAG-873 spp.* (log2FC = -25.205; adj. *p* = 1.60e-17), and *Holdemanella* spp. (log2FC = -12.099; adj. *p* = 3.73e-5). On the other hand, CD resulted in a significant increase in the abundance of *Akkermansia* spp. (log2FC = 3.183; adj. *p* = 0.015), *CAG-873* spp. (log2FC = 19.923; adj. *p* = 9.64e-10), *Hungatella* spp. (log2FC = 2.205; adj. *p* = 0.040), *Lachnospiraceae_UCG-008* spp. (log2FC = 2.709; adj. *p* = 0.026), and *NK4A214_group* spp. (log2FC = 8.969; adj. *p* = 0.040), and a decrease of *Frisingicoccus* spp. (log2FC = -23.318, adj. *p* = 2.96e-13), *Megasphaera* spp. (log2FC = -14.384; adj. *p* = 4.43e-05), *Synergistes* spp. (log2FC = -25.409; adj. *p* = 1.67e-15) and *Tyzzerella* spp. (log2FC = -10.946; adj. *p* = 0.006) (Figure 4B).

### Fecal SCFAs and MCFAs profiles

The abundances of fecal SCFAs and MCFAs before and after both dietary interventions are reported in Table 1. In AD group, a significant increase (*p* < 0.05) was observed in the abundance of acetic (+0.7%), isobutyric (+30.1%), 2-methylbutyric (+64.2%), and isovaleric (+22.5%) acids. On the other hand, CD resulted in increased levels of isobutyric (+71.4%), 2-methylbutyric (+116.2%), isovaleric (+99%), and valeric (+21.4%) acids, and a reduction of butyric (-31.6%) and hexanoic (-66.4%) acids.

**TABLE 1:** SCFAs and MCFAs variation according to the dietary interventions.

	pre-AD	post-AD	P-value	pre-CD	post-CD	P-value
**SCFAs (%)**						
Acetic	52.38 (7.10)	52.74 (8.18)	0.018	52.17 (10.32)	51.01 (15.79)	0.965
Propionic	13.80 (17.24)	15.83 (3.05)	0.204	15.08 (4.29)	13.84 (4.53)	0.295
Butyric	12.19 (5.33)	14.91 (4.31)	0.762	19.06 (9.04)	13.03 (5.54)	0.018
Isobutyric	1.73 (1.46)	2.25 (0.85)	0.016	1.47 (1.33)	2.52 (1.51)	0.032
2-Methylbutyric	1.23 (1.19)	2.02 (0.79)	0.023	0.99 (1.31)	2.14 (1.36)	0.016
Isovaleric	1.42 (1.19)	1.74 (0.86)	0.008	1.02 (1.13)	2.03 (1.28)	0.032
Valeric	2.71 (0.82)	2.99 (0.69)	0.076	2.38 (1.41)	2.89 (2.02)	0.023
**MCFAs (%)**						
Hexanoic	0.76 (1.19)	1.03 (1.19)	0.762	1.16 (1.54)	0.39 (0.65)	0.026
Isohexanoic	0.02 (0.02)	0.02 (0.02)	1.000	0.02 (0.04)	0.01 (0.01)	0.050
Heptanoic	0.01 (0.15)	0.12 (0.21)	0.314	0.08 (0.14)	0.05 (0.10)	0.102
Octanoic	0.01 (0.01)	0.01 (0.02)	0.623	0.01 (0.01)	4.3e-3 (0.01)	0.295
Non-anoic	2.7e-3 (3.9e-3)	4.1e-3 (6.2e-3)	1.000	3.5e-3 (1.4e-3)	3.7e-3 (6.9e-3)	0.227
Decanoic	2.6e-3 (3.7e-3)	1.8e-3 (1.8e-3)	0.285	3.5e-3 (5.6e-3)	2.6e-3 (2.4e-3)	0.239
Dodecanoic	6.1e-3 (0.01)	5.1e-3 (8.4e-3)	0.466	4.9e-3 (0.01)	0.01 (0.01)	0.930

Data are presented as median percentage (interquartile range, IQR). AD, ancient-wheat diet; CD, control-wheat diet; SCFAs, short chain fatty acids; MCFAs, medium chain fatty acids.

### PICRUST analysis

**FIGURE 5:**
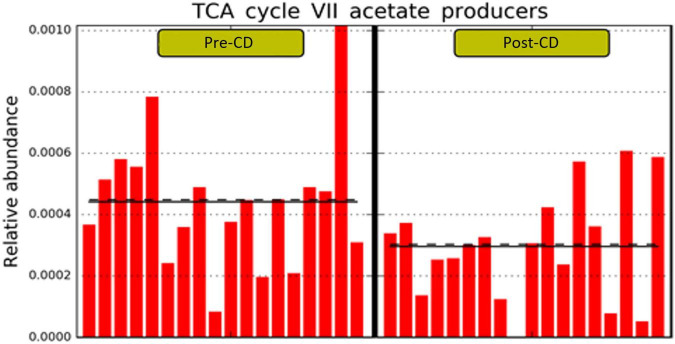
Statistically significant different predicted pathway with LDA score > 2.0 between pre- and post-CD. LDA, linear discriminant analysis; CD, control wheat diet.

Finally, the PICRUSt2 (phylogenetic investigation of communities by reconstruction of unobserved states) predictive metabolism approach (19) was used on the 16S rRNA gene sequencing data to assess functional and metabolic changes of microbial communities after both dietary interventions. Based on MetaCyc metabolic pathway database,^1^ we found no metabolic differences after AD, while CD determined a significant reduction of the pathway PWY-7254 (TCA_cycle_VII_acetate_producers_) (LDA score > 2.0; *p* = 0.046) (Figure 5).

## Discussion

Ancient wheat varieties have gained increasing interest for their nutritional profile but especially for the supposed ability to reduce the risk of chronic diseases compared with modern wheat. Nevertheless, the mechanisms by which they confer protective effects on human health and their potential influence on GM composition are still unclear. In this scenario, we have conducted randomized, double-blind crossover trial to explore if a replacement diet with pasta made with ancient wheat could modify the GM composition and production of bacterial metabolites, such as SCFAs and MCFAs, compared to a replacement diet with pasta made from modern wheat.

In agreement with Saa and colleagues (20), who found no changes in the GM composition of healthy volunteers after a 3-month diet with Khorasan wheat or whole durum wheat, we observed that the two interventions did not significantly modify either the structure or the microbial diversity. In addition, Carroccio and colleagues recently found no statistically significant differences in alpha diversity indices after a 30-day intervention with ancient and modern wheat-based foods (21). These findings are not surprising, since a considerable GM stability and resilience have also been demonstrated with other nutritional interventions (22).

Some significant variations, on the other hand, have been reported at genus level. The ancient wheat pasta administration determined a significant increase of *Erysipelatoclostridium* spp. and a significant decrease of the genera *[Bacteroides]_pectinophilus_group, CAG-873* and *Holdemanella*. In this regard, Martin-Gallausiaux and colleagues have documented that members of genus *Erysipelatoclostridium* are bacteria producing butyrate, a crucial SCFA with renowned and potent anti-inflammatory properties (23). In addition, the *[Bacteroides]_pectinophilus_group* is well known as a genus constituted by complex carbohydrate degrading bacteria (24). As to *Holdemanella* spp., Pujo and collaborators showed its anti-inflammatory properties in ulcerative colitis patients (25).

Regarding the control pasta intervention, it determined a significant increase of *Akkermansia* spp., *CAG-87* spp., *Hungatella* spp., *Lachnospiraceae_UCG-008* spp., and *NK4A214_group* spp. and a significant decrease of the genera *Frisingicoccus*, *Megasphaera*, *Synergistes*, and *Tyzzerella*. According to recent findings, *Akkermansia* spp. may exert anti-inflammatory properties in the intestinal tract, and enhances the intestinal barrier integrity and function (26); on the contrary, the genus *Hungatella*, which includes trimethylamine-producers members, have been associated with cardiovascular diseases (27). Also increased levels of *Lachnospiraceae_UCG-008* spp. *Tyzzerella* spp. have been associated with chronic intestinal inflammation (28, 29), while a high abundance of *Frisingicoccus* spp. have been reported in patients affected by Parkinson’s disease (30).

Furthermore, we evaluated the production of bacteria-derived metabolites, by analyzing fecal SCFAs and MCFAs profiles. As is well known, the SCFAs are mainly produced by bacterial anaerobic fermentation of indigestible polysaccharides and can either be absorbed by the colonic epithelium providing energy or enter the bloodstream and play a relevant role in the regulation of fatty acids, glucose, and cholesterol metabolism (31). In addition, they mediate the beneficial effects of dietary fiber, i.e., protection against cardiovascular disease, colorectal cancer, obesity, and diabetes (32). According to recent studies, branched SCFAs such as isovaleric and isobutyric acids may also exert a beneficial influence on host metabolism and especially immunity, preventing inflammatory diseases and metabolic disorders (33). In our study, the ancient wheat pasta intervention caused a significant increase of acetic, isobutyric, 2-methylbutyric and isovaleric acids. These findings are in agreement with those of the, previously reported, study of Saa and colleagues documenting an increased SCFAs’ levels after a 3-month Khorasan based diet in healthy individuals (20). In addition, we have previously documented increased SCFAs’ production in patients with fibromyalgia syndrome who consumed Khorasan wheat (34). Rios-Covian and colleagues, on the other hand, reported an association between branched SCFAs and lower fiber consumption, hypothesizing that the reduced availability of fermentable carbohydrates by the GM promoted a shift to increased protein fermentation (35). Regarding control modern wheat pasta, the intervention resulted in increased levels of isobutyric, 2-methylbutyric, isovaleric and valeric acids, and a reduction of hexanoic and butyric acids. Notably, butyric acid is one of the most abundant SCFAs produced by intestinal bacteria that, being (i) a potent activator of both G protein-coupled receptors (35) and peroxisome proliferator-activated receptors (36) and (ii) a potent inhibitor of histone deacetylases (37), exerts multiple beneficial effects such as the modulation of visceral sensitivity and immune responses, and the expression regulation of some critical genes (36).

Finally, we used the PICRUSt2 to assess eventual functional change of microbial communities after both dietary interventions. While no metabolic differences were found with the ancient wheat pasta, a significant reduction of the TCA_cycle_VII_acetate_producers_ pathway was observed with the modern wheat pasta. The TCA (tricarboxylic acid cycle) pathway is usually used as a reference for its central role in energy generation. Indeed, it is present in most aerobic living organisms that use this cycle to generate NADH and FADH2 and utilize these molecules as the reductive potential required for ATP synthesis during oxidative phosphorylation (37). In contrast to our findings, Carroccio and colleagues showed that the GM ability to metabolize carbohydrates increased only after a diet containing ancient grain products, and not with a modern wheat-based diet (21).

Surely our study has some limitations, such as the restricted number of participants, the limited study duration, and the low taxonomical resolution of 16S rRNA sequencing. Furthermore, it was not possible to consider any lifestyle changes that might have affected the study, even though participants were under strict instruction to maintain their usual lifestyle patterns while consuming ancient and modern wheat pasta. We are aware that 8 weeks of intervention is a limited period and permits only the suggestion of the possible interpretation of the results. However, the present study shows different strengths, including the double-blind randomized cross-over design, the good compliance of participants to the assigned diets, and finally the fact that data on ancient wheat consumption and GM modifications are currently very uncommon.

In conclusion, a short-term replacement diet with ancient and modern wheat pasta did not modify either the structure or the microbial diversity of gut microbiota. However, both interventions determined significant changes in GM composition at the genus level. As to bacteria-derived metabolites, the administration of ancient wheat pasta resulted in a greater beneficial impact on anti-inflammatory SCFAs. Anyway, further studies with larger number of subjects and longer periods of dietary intervention are needed to confirm these preliminary results.

## Data availability statement

The data presented in this study are deposited in the NCBI Gene Expression Omnibus (GEO) repository, accession number GSE206807.

## Ethics statement

The studies involving human participants were reviewed and approved by Ethics Committee of the Tuscany Region, Careggi University Hospital. The patients/participants provided their written informed consent to participate in this study.

## Author contributions

SBa, MD, AA, and FS conceived and designed the study and drafted the manuscript. SBa and LC acquired experimental data of microbiota. MD, GP, BC, and FS were involved in subjects’ enrolment. SBa, MP, and GP performed gas chromatography-mass spectrometer analysis. LD and MR performed bioinformatic and statistical analysis of microbiota. SBa, MD, GP, BC, LC, LD MP, MR, and GB analyzed and interpreted the data. SBe, AA, and FS critically revised the manuscript. All authors contributed to the article and approved the submitted version.

## Footnotes

1
www.metacyc.org
